# Gene structure and expression of serotonin receptor *HTR2C *in hypothalamic samples from infanticidal and control sows

**DOI:** 10.1186/1471-2202-13-37

**Published:** 2012-04-02

**Authors:** Claire R Quilter, Meenashki Bagga, Ahmad Moinie, Fatima Junaid, Carole A Sargent

**Affiliations:** 1 University of Cambridge, Department of Pathology, Tennis Court Road, Cambridge CB2 1QP, UK; 2 University of Oxford, John Radcliffe Hospital, Oxford OX3 9DU, UK; 3 University of Cambridge, Department of Clinical Medicine, Addenbrookes Hospital, Cambridge CB2 0SP, UK

**Keywords:** Maternal behaviour, Serotonin receptor, Gene structure, RNA editing

## Abstract

**Background:**

The serotonin pathways have been implicated in behavioural phenotypes in a number of species, including human, rat, mouse, dog and chicken. Components of the pathways, including the receptors, are major targets for drugs used to treat a variety of physiological and psychiatric conditions in humans. In our previous studies we have identified genetic loci potentially contributing to maternal infanticide in pigs, which includes a locus on the porcine X chromosome long arm. The serotonin receptor *HTR2C *maps to this region, and is therefore an attractive candidate for further study based on its function and its position in the genome.

**Results:**

In this paper we describe the structure of the major transcripts produced from the porcine *HTR2C *locus using cDNA prepared from porcine hypothalamic and pooled total brain samples. We have confirmed conservation of sites altered by RNA editing in other mammalian species, and identified polymorphisms in the gene sequence. Finally, we have analysed expression and editing of *HTR2C *in hypothalamus samples from infanticidal and control animals.

**Conclusions:**

The results confirm that although the expression of the long transcriptional variant of *HTR2C *is raised in infanticidal animals, the overall patterns of editing in the hypothalamus are similar between the two states.

Sequences associated with the cDNA and genomic structures of *HTR2C *reported in this paper are deposited in GenBank under accession numbers FR720593, FR720594 and FR744452.

## Background

The 5-hydroxytryptamine (serotonin) receptor 2C (HTR2C) is one of a family of receptors that is involved in coordinating the intracellular responses to serotonin in the mammalian nervous system [1,2]. This particular protein couples the binding of ligand to the surface receptor domain with the mobilization of intracellular calcium via G-protein coupling and activation of protein kinase pathways. Although the best understood activities of HTR2C are those elicited via activation of phospholipase C (PLC), there is evidence that other signalling cascades such as phospholipase D and A2 pathways can be stimulated [3].

The full-length HTR2C receptor is a seven transmembrane domain protein, and has a phosphorylation site in the cytoplasmic tail. It is a target for drug agonists and antagonists used to treat a variety of physiological and psychiatric conditions; the structures of the receptor-ligand complexes have been computationally predicted based on successes with other G-protein coupled receptors such as the β2 adrenergic receptor and D2 dopamine receptor [4]. The exon encoding the second intracellular loop, which interacts with G-proteins, is subject to a complex series of mRNA editing events representing 24 potential isoforms of the protein [5,6]. The potential contribution of each amino acid change to receptor activity *in vivo *has been tested by studies using PLC activation as a reporter [3,7,8]. An additional level of protein regulation occurs through a cryptic internal splice site in the third coding exon which results in premature termination of the open reading frame. In rodents, both the splice junction usage and the mRNA editing are thought to be regulated via non-coding RNAs. In particular the snoRNAs of the *Snord115 *locus, syntenic with the Prader-Willi Syndrome locus on human chromosome 15 [9], is implicated in mouse models [10,11]. However, the role of the syntenic locus in humans has been controversial, and more recent analyses have revealed mir-22 as a regulator of not only *HTR2C *mRNA expression, but also of other candidate genes for panic disorder [12]. Unfortunately, this latter study does not report which isoform(s) of the transcripts are affected. Alternative experimental evidence suggests that the intramolecular dsRNA loops formed by the full length *HTR2C *RNA transcript are sufficient to promote editing via the ADAR proteins, and that inhibition of the secondary structures through the introduction of an RNA binding peptide opens the possibility of novel, editing-directed therapies in the future [13]. It is proposed that this intricate series of transcriptional modifications allows fine-tuning of the serotonergic and dopaminergic pathways in response to neuronal cell types, stress and circulating neurotransmitter concentrations. All act to down-regulate the constitutive activity of the unedited, full-length form of the protein. Furthermore, detailed sequencing analysis of the extent of editing in the mouse brain confirms that it varies according to the stage of development from the embryo through to the adult [14]: thus, the regulatory mechanisms are highly responsive both spatially and temporally.

Advances in our understanding of the regulation of expression of the HTR2C receptor through transcriptional control provide additional insights into the physiological roles of the protein. HTR2C is central to a number of homeostatic and behavioural responses. These include control of circadian rhythms, leptin regulation of appetite, energy expenditure and bone mass, anxiety, depression, and the control of the release of other hormones such as pro-opiomelanocortin and prolactin from the pituitary gland [15,16]. These serotonin responses are elicited mainly via those HTR2C receptors present in the limbic system and the hypothalamus. In contrast, using a variety of agonists and antagonists both HTR2C and HTR2A receptors in the prefrontal cortex of the rat brain have been shown to be required for locomotor activity [17].

Overall, *HTR2C *is an attractive candidate to study in animal models of behavioural responses, particularly aggressive behaviours, and has been investigated at the genetic and functional levels in a range of species including rat, mouse, dog and chicken [18-23]. In human neurodevelopmental disorders such as attention deficit hyperactivity disorder (ADHD) [24] and psychiatric disorders such as puerperal psychosis [25] there is a modest association with *HTR2C *specific haplotypes, but many genetic association studies in humans are inconclusive or contradictory. More detailed recent analyses looking at the levels of gene expression, protein activity and the potential isoform composition in different brain subregions with relevance to genotypes and behaviours support a role for editing, not just polymorphism, at the *HTR2C *locus, which could explain why genetic association studies have been inconclusive [26-30]. The argument for a central role for HTR2C receptor in psychoses, anxiety disorders and stress based on the biological and functional evidence remains strong, and has been reviewed by Drago and Serretti [31].

Our previous studies have identified some modest changes in *HTR2C *transcriptional levels in the hypothalamic regions of sows showing infanticidal aggressive behaviours when compared with their non-aggressive counterparts [32]. In a parallel study to identify QTL contributing to the behaviour, we identified at least two peaks on the porcine X chromosome, one of which lay on the long arm [33]. This locus on the long arm of the X chromosome has been independently confirmed in a porcine resource population created from European White Duroc crossed with Chinese Erhualian [34]. Of those genes already implicated in human mood disorders and rodent behaviours, *HTR2C *is both a functional and positional candidate for further investigation, as it maps to porcine Xq22-23.

In this paper we describe the analysis of the porcine gene at both the genomic and cDNA levels. The open reading frame of the receptor has been scanned for polymorphisms, and the mRNA editing of the transcript investigated in the hypothalamic samples from sows showing maternal infanticide and their matched controls. Overall, we found evidence for increased mRNA expression but not for significant differences in the editing profiles in the hypothalamus of infanticidal animals.

## Results

### Gene structure, and polymorphism

The *in silico *analysis combined with the genomic PCR and cDNA PCR experiments (primers in Table 1) confirm that the porcine gene has a similar structure to the human gene, with conservation of the three non-coding 5'UTR exons and all four of the exons encoding the serotonin receptor and 3' UTR at the genomic level. In addition, the region immediately upstream of the putative serotonin transcriptional start site suggests that there is conservation of regulatory sequences between the pig, human and other mammalian species: this region contains a predicted CpG island, and is likely to contain other motifs for transcriptional control. This region was not included for sequence analysis in our study. Following sequencing of the cDNA fragments, the 5'UTR was found to be homologous to exons 1, 2, and part of exon 4 from human transcript ENST00000371951 and a sequence equivalent to exon 3 was not present in the cDNA from the pooled brain sample. The full length porcine transcript is deposited under accession number FR720593.

**Table 1:** Primers used in this study

Primers for HTR2C amplification				
	forward	reverse	template	size (bp)
Exon 1	TTTAGCCCAAGAGAGCGATG	TAGGGTCTGGGCTAGCAATG	genomic	637
Exon 2	TTCTGGCACTAATGAATATCAGC	CATGGTAAGTCAATAACGTCAAATG	genomic	315
Exon 3	CTGCATCCCCATTATGAGC	TGGACCTTTCCTTGCATCTC	genomic	300
Exon 4	AAATGAAAAATCTATCCTCTTTGC	GGCAGATTGAGGCAAATATAGC	genomic	413
Exon 5A	ACTCTGGGGACAGGAGGAAG	CTGCCATGATGACGAGAATG	genomic	400
Exon 5B	ATGGTGGACGCTTCAAATTC	TGCGCACATTCAATTACCTC	genomic	363
Exon 6	GCCCTAGAAAAGGCAAAATG	TAGCCGCTGCAATTCTACTG	genomic	463
Exon 7A	ACCCTTCCGTGTGCTGTAAC	CAAGCCTTCCCACAAAGAAC	genomic	519
Exon 7B	GAAAGCGTCGAAAGTCCTTG	TCAACATTTTTGCATCGAAC	genomic/cDNA	695
HTR2CX1-2	TTTAGCCCAAGAGAGCGATG	TTGAAGGATGGGGATTCTTG	cDNA	653
HTR2CX2-5	GTAGGCCAAGAATCCCCATC	ACCAATAGGCCAATCAGGTG	cDNA	263
HTR2CX4-5	CATGGTGAACCTGAGGAAAG	GCAGAGACAGTGGCATGA	cDNA	335
HTR2CX5-7	CATGCCACTGTCTCTGCTTG	TGTTGTTGACGAACACCTTG	cDNA	299/204
HTR2CNR		TTGCCCAAACAATAGCAATC	cDNA	
HTR2CL	TTCTACAGCGTCCATCATGC	CACCTAAAGAAATTGCCCAAAC	qRT-PCR	143
HTR2CS	TGTCATGCCACTGTCTCTGC	ACACCGATCCAGCGAAATAG	qRT-PCR	145

A list of primers used for PCR amplification of *HTR2C *exons from both genomic DNA, and cDNA prepared from a transcript pool. Primers were selected following comparison of human and porcine reference sequences to define the putative coding regions of the pig genome

Sequencing of each of the exons in the selected pig lines revealed no polymorphisms in exons 1 to 6, although several non-coding polymorphisms were detected in introns 2, 3 and 4. Four polymorphisms were detected in exon 7, of which three are silent, and the fourth is a conservative replacement at amino acid position 275 (S to N). All of the polymorphisms are deposited under accession number FR744452, which incorporates the identified exons and their immediate flanking sequences.

The cDNA sequences from pooled brain were compared against the consensus genomic sequences to look for further coding region polymorphisms. No novel variants were identified.

### Alternative splicing and mRNA editing

Amplification of the open reading frame at the level of cDNA showed that two major products are obtained from *HTR2C *transcription in pig. On sequencing, the longer product was found to correspond to the open reading frame of the entire functional receptor, but the shorter PCR product represented the transcript that uses an internal splice site in exon 6. The cDNA sequence indicated that exon 6 in the mRNA is not fully concordant with the genomic sequence at five positions. These are analogous to the bases modified in rodents and humans by RNA editing, with five consecutive A**→ **G conversions. This changes the conceptual amino acid sequence A***I***R***N***P***I***E to A***V***R***G***P***V***E when full editing is observed.

### RNA editing in individual animals

Two approaches were used to estimate editing in the infanticidal and control animals. Firstly, the sequence traces were used to estimate the extent of editing at each position based upon the relative peak heights [35]. The results were compared at each position between the paired sample set, and the *p *values for differential editing calculated per site using a paired Student's *t*-test, with 8 degrees of freedom. No significant differences were observed between the infanticidal and control sets of animals. Similarly, no statistically significant differences were observed between the Large White (LW) v Hampshire X Large White (HxLW) pairs, although there was a trend for the HxLW to show slightly elevated total editing across the five sites (Table 2 and Table 3)

**Table 2:** Summary of editing data

	Site A		Site B		Site C'		Site C		Site D	
Subcloning *	**Mean ± SEM**	**p**	**Mean ± SEM**	**p**	**Mean ± SEM**	**p**	**Mean ± SEM**	**p**	**Mean ± SEM**	**p**

Pairs 1-9 I Pairs 1-9 C	52.8 ± 4.5 53.1 ± 4.2	0.955	31.9 ± 4.5 30.1 ± 3.9	0.794	8.44 ± 1.8 8.22 ± 1.7	0.943	53.9 ± 4.5 51.2 ± 4.2	0.642	68.3 ± 6.1 70.0 ± 2.9	0.753
Sequence Traces**										
Pairs 1-9 I Pairs 1-9 C	50.9 ± 4.4 43.1 ± 2.8	0.121	40 ± 4.5 32.8 ± 2.7	0.130	14.8 ± 2.8 11.2 ± 1.9	0.282	35.6 ± 5.8 37.1 ± 2.8	0.769	77 ± 3.1 78 ± 2.6	0.749

Data is provided per edited site, using the conventional site labelling of A, B, C', C, D, respectively along the coding sequence. All nine pairs of animals are included: I, infanticidal animal(s); C, control animal(s). *data collected from sequencing subcloned products. Minimum of 40 clones sequenced per sample. Range 40-48, mean 44 except for pairs 2 (60/48) and 3 (59/59) (I:C, respectively). **based on relative peak heights from sequence traces.

**Table 3:** Average editing across all edited sites, for all pairs and by breed background

	% Average editing, all sites trace data	% Average editing, all sites cloned data
**I (n = 9)**	43.6 ± 3.5	42.4 ± 3.7
**C(n = 9)**	40.4 ± 1.8	42.7 ± 2.4
**LW I (n = 4)**	43.8 ± 6.3	41.1 ± 8.8
**LW C (n = 4)**	38.1 ± 3.5	38.8 ± 5.4
**HxLW I (n = 4)**	44.5 ± 5.6	43.1 ± 2.4
**HxLW C (n = 4)**	42.2 ± 2.9	46.4 ± 2.6

The average editing for each of the sets of data is summarised for all pairs and broken down according to breed background. LW (Large White) and HxLW (Hampshire x Large White)

Subclones were sequenced from each of the 18 animals to determine the combination of edited sites on independent molecules. The statistical comparison based on matched pairs indicated that no single site or specific edited isoform reached significance in the infanticidal compared with the non-infanticidal group. Similarly, no sites reached significance when the LW animals were compared with the HxLW animals, but the trend agreed with that observed with the trace ratio results: slightly elevated editing was observed in the HxLW compared with the LW animals (Table 4). As expected from analysis of the cloned sequence data, there was no significant difference at the amino acid level, either by individual site, or by conceptual translations of the sequences across all edited sites.

**Table 4:** Editing by site and breed background

	% Per site, trace data				% Per site, cloned data			
	**LW I**	**LW C**	**HxLW I**	**HxLW C**	**LW I**	**LW C**	**HxLW I**	**HxLW C**

**A**	53.0 ± 5.9	38.5 ± 4.5	51.3 ± 8.3	46.5 ± 3.9	52.0 ± 10.3	48.3 ± 7.5	53.5 ± 3.8	57.3 ± 6.0
**B**	41.5 ± 7.1	28.3 ± 4.2	40.8 ± 8.2	36.8 ± 3.5	28.0 ± 10.0	24.3 ± 7.6	27.3 ± 4.6	36.5 ± 2.9
**C'**	15.8 ± 5.6	11.0 ± 1.1	14.8 ± 3.6	11.5 ± 4.2	10.3 ± 3.4	6.25 ± 2.8	8.75 ± 1.8	9.0 ± 2.5
**C**	31 ± 11.4	37.8 ± 6.6	38.8 ± 7.8	36.0 ± 1.6	52.3 ± 8.05	48.0 ± 9.1	53.3 ± 6.8	55.3 ± 4.0
**D**	77.5 ± 5.7	76.3 ± 5.1	77.0 ± 4.8	80.3 ± 3.2	63.0 ± 13.8	67.5 ± 5.4	72.5 ± 3.9	73.8 ± 3.7

Large White pairs (n = 4) compared against Hampshire x Large White pairs (n = 4) using cloned sequence analysis or peak ratio heights in sequence traces. Results in Table 4 are given as the mean ± SEM. No statistically significant differences were observed between the groups based on different breed backgrounds

### Analysis of *HTR2C *transcript expression levels

Quantification of the level of *HTR2C *transcript in infanticidal and control animals used different sets of primers to identify both the total amount of transcript (HTR2CS) and the long isoform (HTR2CL) only. Comparison of the two groups with each primer pair (Table 5) (IvC) showed a trend for infanticidal animals to express slightly more transcript than the controls, but this was not statistically significant (*p *= 0.3).

**Table 5:** Quantitative PCR results for different HTR2C primers by breed background

	Relative quantity HTR2CL	Relative quantity HTR2CS
**I (n = 9)**	26.98 ± 3.66	51.53 ± 4.40
**C(n = 9)**	24.71 ± 3.21	44.03 ± 3.95
**LW I (n = 4)**	32.77 ± 3.10	54.37 ± 7.60
**LW C (n = 4)**	29.37 ± 3.70	51.92 ± 6.00
**HxLW I (n = 4)**	20.24 ± 2.30	45.41 ± 5.80
**HxLW C (n = 4)**	18.53 ± 1.0	38.30 ± 3.30

Quantitative PCR was carried out using primer pairs HTR2CL (specific for the long isoform) and primer pair HTR2CS (both isoforms) using PSMD2 primer pairs as a control described in Quilter *et al. *2008. Results from all pairs (n = 9) and from Large White pairs (n = 4) or Hampshire x Large White pairs (n = 4) in Table 5 are given as the mean ± SEM. No statistically significant differences were observed between the infanticidal and control groups within breed, but the LW results were statistically different from the HxLW results (*p *= 0.001) for the expression of the long isoform

The results from the two breeds of animals were compared to determine whether breed or state had a larger impact on *HTR2C *expression. In the first comparison, a three-way ANOVA was used to look at the interaction of 'breed' (LW or HxLW), 'state' (I or C) and all qRT-PCR expression data (both HTR2CS and HTR2CL). In this, breed was found to be highly significant (*p *= 4.2 × 10^-7^), expression levels were significant (*p *= 0.001), but behavioural trait (*p *= 0.27) and interactions were not significant. Next, the data from each of the qRT-PCR comparisons were individually tested using a two-way ANOVA in which 'breed' and 'state' were considered independently or for interaction. The results showed greater significance for 'state' (*p *= 0.001), than for 'breed' (*p *= 0.37), or breed with state interaction (*p *= 0.76) with the long isoform (HTR2CL) primer pair, but with less significant results for the total amount of transcript detected using HTR2CS (state, *p *= 0.08; breed, *p *= 0.43; breed with state interaction, *p *= 0.70).

## Discussion

This paper describes the structure of the porcine HTR2C transcripts as determined using RT-PCR with cDNA prepared from a pool of mRNA isolated from brain tissue. The full-length open reading frame sequence is most analogous to human transcript ENST00000276198 (reference sequence NM_000868). Although exon 3 of human transcript ENST00000371951 is conserved at the genomic level, we assume that this isoform and its orthologues have limited expression in humans and in other mammals, and may be confined to specific neurons in the brain. Two major splice sites define the 3' end of exon 6: both are canonical splice junctions. The first splice junction generates the short form of the protein, which leads to premature truncation of the polypeptide, the loss of transmembrane domains 6 and 7 and the intracellular region thought to be essential for G protein binding. The position of the splice site is analogous to that in human and rodent genes: however, in each species the truncation of the polypeptide occurs at a unique position within the final coding exon. The second splice junction generates the full length transcript, but the complex post-transcriptional editing mechanism generates a range of different isoforms that modify the ability of the receptor-ligand complex to initiate and perpetuate phospholipase dependent intracellular signalling pathways.

Only one of the four polymorphisms in the transcript was identified in the protein coding region in this study (S275N). This amino acid lies in the linker region between transmembrane domains 5 and 6, at a site not previously implicated in functional changes to HTR2C proteins. Any impact of this change on downstream signalling pathways is unknown.

For each of the two breed backgrounds in this study, three of the four pairs show upregulation of *HTR2C *gene expression in infanticidal animals. This is consistent with previously published results [32] using array probe data and qRT-PCR primers from the final coding exon, where a trend for increased expression of *HTR2C *in infancticidal animals was noted. In this paper, we have looked at both the relative quantity of the longer and shorter isoforms, and the potential impact of editing on protein expression between the two groups. Our results support increased mRNA expression in the hypothalamic samples from the infanticidal animals. It is interesting that there is a more significant difference between the two behavioural states for the relative quantity of the longer isoform since this is potentially the most active variant of the receptor, which can subsequently be modified through the action of the editing process. However, there is no statistically significant difference in the qualitative data we have obtained for the degree of mRNA editing.

One obvious limitation to this study is the small number of available samples from animals of the same genetic background. However, the implication of the three-way ANOVA is that breed may also be relevant in determining the overall expression of the receptor, and the observed breed specific differences in the incidence of abnormal maternal behaviour support such a complex, multifactorial model.

Although qualitative changes have been described in a number of other studies relating to both human and rodent populations with presumed genetic or drug-induced behavioural differences, it should also be noted that most of these have analysed different regions of the brain from that described in this paper. The distribution of *HTR2C *isoforms is remarkably diverse and dependent upon the neuroanatomical region under investigation [14]. In general, the evidence for editing patterns influencing behaviour has been replicated in studies of material collected from the (pre)frontal cortex in both humans [30] and rat [17,18,20]. All of these papers support a role for the gene in anxiety related behaviours, including some forms of aggression. In contrast, using whole brain mRNA from a murine model of Lesch-Nyhan Disease [19], Bertelli *et al. *showed that although the level of the *HTR2C *transcript is elevated, the pattern of editing per site is not affected, which is entirely consistent with our observations here. Again, this raises the issue of whether significant changes are confined to specific sub-sets of neurones within the brain, and that the explicit role of editing can only be fully understood with more precisely dissected samples.

To date, the only positive correlation between behaviour and serotonin receptors within the hypothalamus is with HTR2A. This receptor is de-sensitized preferentially following prolonged glucocorticoid exposure [36]. This implicates the HTR2A receptor in the dampened HPA axis response following prolonged stress, and specifically the action of HTR2A bearing neurones within the paraventrical nucleus (PVN). However, the HTR2C receptor did not seem to be directly involved in this model.

Although *HTR2C *has been implicated in genetic studies of human behavioural abnormalities, many of these have focussed on using well described polymorphic markers, either singly or as haplotypes. Results are contradictory, and currently the role of this specific receptor remains inconclusive. At the DNA level, the locus is complex, since the intronic regions of the gene also harbour loci for non-coding micro-RNA (miRNA) species. Such RNA molecules are known to be expressed at a high level in the brain, and regulate the expression of downstream targets. In addition *HTR2C *has itself been identified as a potential target for miRNAs encoded elsewhere in the genome. With such molecular and regulatory complexity, clarification of the evidence will require further experimental work before the *HTR2C *region of the X-chromosome can be excluded from the list of candidate loci involved in the control of behaviours. Firstly, the contributions of *HTR2C *gene expression in different neuroanatomical regions of the porcine brain need to be analysed more precisely, and, secondly, we need more understanding of how the associated miRNAs regulate expression patterns of both the *HTR2C *locus, and other brain expressed genes.

## Conclusions

This study supports elevated levels of gene expression in infanticidal animals, but has not yet provided direct evidence for the involvement of *HTR2C *editing in the hypothalamus. Further work to investigate the distribution of the respective splice variants in cross-sections representing more detailed subregions of the brain, combined with a fuller understanding of the biological functions of the encoded miRNAs within the gene are still required. Such in-depth analysis may help to explain why there are recurrent data that implicate this locus in behavioural phenotypes in the absence of consistent genetic correlates.

## Methods

### *In Silico *identification of the porcine HTR2C open reading frame from genomic sequences

The porcine sequences homologous to the human gene were identified from the nucleotide databases using BLAST. The longest human cDNA sequence (Ensembl transcript HTR2C-001: ENST00000371951) has seven exons of which exons 4-7 encode the functional protein. This transcript was compared against the EST, htgs, nr, genome and trace archives at NCBI. The 5' untranslated region of the transcript (exons 1-3) was identified in *Sus scrofa *chromosome X clone CH242-371K6 (accession number CU606877), the first two coding exons (exons 4, 5) were identified in *Sus scrofa *chromosome X clone CH242-408J11 (accession number CU207399) and the last two coding exons (exons 6, 7) were identified in *Sus scrofa *chromosome X clone CH242-135K13 (accession number CU856526) all of which map to the long arm of the pig X chromosome (Xq22-Xq23: 98-99 Mb, based on the Sscrofa9 build of the pig genome: http://www.ensembl.org/Sus_scrofa/Info/Index[37]).

The coding exons were also identified in genomic and cDNA sequences from the trace archives using the human and pig ESTs as the query sequences. Multiple sequence reads were aligned to confirm the consensus for each coding exon and the associated intronic flanks. All consensus sequences were reconfirmed against the human genome at both the nucleotide and protein coding levels. Primers were designed from the consensus sequences with homology to the human transcript ENST00000371951 (the longest Ensembl coding transcript) 5' UTR (exons 1, 2, and 3), coding and 3' UTR (exons 4, 5, 6, and 7) using the program, Primer3, v.0.4.0 (http://frodo.wi.mit.edu/primer3/[38]).

### Confirmation of exonic structure by PCR. Polymorphism analysis of genomic DNA

Each predicted exon was amplified from porcine genomic DNA from four pig sample collections representing pure-bred Landrace, pure-bred Large White, and two Duroc synthetics then sequenced as below. Six individuals were used per breed. All PCR reactions were carried out on 10 ng cDNA in 10 μl reaction volumes using HotStarTaq DNA polymerase (Qiagen, Hilden, Germany). Reactions were carried out on a GeneAmp PCR 9700 System machine according to the following conditions: 95°C for a 15 min hot start, then 95°C for 30 s (denaturation), 60°C for 30 s (annealing), 72°C for 1 min (extension) for 35 cycles and finally incubation at 72°C for 10 min.

The amplified PCR products were prepared using ExoSAP-IT (USB, US) according to the manufacturer's instructions and sequenced with BigDye Terminator v3.1 Cycle Sequencing RR-100 (Applied Biosystems). All sequencing reactions were run on an Applied Biosystems 3730 × l DNA Analyzer by Source BioScience LifeSciences (Cambridge, UK). Traces were manually edited then compared using the Sequencher 4.9 software (Gene Codes Corporation, US) for identification of SNPs.

### Confirmation of alternative splicing of transcripts and exon usage from brain cDNA

Primers were designed from the predicted transcript and used to amplify the open reading frame in fragments to confirm the sequences, then as a single major product from cDNA (exons 4 through to exon 7; HTR2CX4-5 forward and exon 7A reverse), using an overlapping primer pair to complete the open reading frame in exon 7 (exon 7B primer pair). The cDNA was prepared from the pool of brain mRNA used as a control for the studies described in [32] using the Reverse Transcription System (Promega, UK). Two products were identified from the exon 4 through to exon 7 PCR, and were sequenced independently.

The primers spanning the region from putative exon 1 to exon 2 (HTR2CX1-2), and exon 2 to exon 5 (HTR2CX2-5) were used to complete the 5' UTR as predicted from the genomic sequence. Primers from exon 1 or exon 2 to exon 5 detected only one band following PCR of cDNA prepared from the brain pool. Sequencing confirmed absence of the predicted exon 3 from the products. All PCRs were carried out on a GeneAmp PCR 9700 System machine, for 35 cycles using HotStarTaq DNA polymerase as above.

### Sequence analysis of genomic DNA and transcripts from sows with and without the maternal aggression phenotype

The mRNA samples from the nine pairs of sows used in the original microarray analysis of hypothalamic transcripts [33] were reverse transcribed to produce cDNA for further investigation of alternative splicing and RNA editing in the maternal aggressive infanticide phenotype. Aliquots were taken for analysis of the alternative products using qRT-PCR. For investigation of mRNA editing, individual PCR products encompassing the edited region of the cDNA were subcloned as described below. Exons were sequenced at the DNA level for individual animals in parallel. No variants or mutations above those found in the original polymorphism screening were identified in the genomic DNA or the cDNA samples.

### Semi-Quantitative analysis of alternative splicing

cDNA prepared as above was subjected to qRT-PCR using two different pairs of primers. The first primer pair HTR2CL only detects the long isoform, as the reverse primer is specific to the exon 6/exon 7 boundary. The second primer pair HTR2CS gives a relative estimate of the total amount of transcript from the gene, as it should detect both the long and short variants equally. All quantitative PCRs were performed on a Bio-Rad iCycler using SYBR green detection (Qiagen) as described previously [32]. Both sets of PCR results were normalised against *PSMD2*, a gene previously identified as being stably expressed in the brain [32,33]. Relative quantities of the *HTR2C *transcripts were calculated using the ΔΔCt method [39]. Data for relative expression values of the long and short forms of the transcript were compared for the effects of state and breed using a two-way ANOVA. Each set of data (long and short isoform) was also analysed independently for relative expression levels with respect to breed background only.

### Analysis of the mRNA editing sites

cDNA from all nine pairs of infanticidal animals and matched controls, plus two control transcript pools, were amplified across the regions in exon 6 that are edited at the mRNA level. Amplification was carried out with primer pair HTR2CX4-5 forward primer and HTR2CNR as the reverse primer. The cDNA was sequenced with HTR2CX5-7 forward primer and run by Source BioScience LifeSciences to confirm the presence of the edited sites. An approximate level of editing was calculated based on the relative peak heights for each base at the edited site [35].

To obtain a more accurate analysis of the degree of modification per site, and the combination of edited sites along each RNA strand with the predicted amino acid sequence, PCR products from individual animals were ligated into pGEM^®^-T Easy vector system (Promega), transformed into *E. coli*, and individual subclones picked for sequencing. A minimum of 40 subclones were picked per animal from a single transformation experiment (efficiency > 10^7 ^cfu/μg DNA per transformation).

The data from the matched infanticidal/non-infanticidal control pairs of animals were analysed using a paired Student's t-test to compare the level of editing at each of the five sites (run via http://www.physics.csbsju.edu/stats/Paired_t-test_NROW_form.html; [40]). ANOVA were run using Octave (website: http://www.gnu.org/software/octave/[41]). The cDNA sequences were also interpreted based on the coding potential, with the combinations of editing leading to the less active isoforms (VSV and VGV) compared against the most active isoform (INI).

## Competing interests

The authors declare that they have no competing interests.

## Authors' contributions

CRQ and CAS provided the project ideas, design, management and interpretation of results. CRQ and MB collected samples, and prepared cDNA for the experimental sections, while AM and FJ carried out PCR experiments, subcloning, sequencing and virtual translations of the products. All authors read and approved the final manuscript.
